# Litter mixing promoted decomposition rate through increasing diversities of phyllosphere microbial communities

**DOI:** 10.3389/fmicb.2022.1009091

**Published:** 2022-11-08

**Authors:** Jiaying Liu, Changjun Ding, Weixi Zhang, Yawei Wei, Yongbin Zhou, Wenxu Zhu

**Affiliations:** ^1^College of Forestry, Shenyang Agriculture University, Shenyang, China; ^2^State Key Laboratory of Tree Genetics and Breeding, Research Institute of Forestry, Chinese Academy of Forestry, Beijing, China; ^3^Research Station of Liaohe-River Plain Forest Ecosystem, Chinese Forest Ecosystem Research Network (CFERN), Shenyang Agricultural University, Tieling, China; ^4^Key Laboratory of Tree Breeding and Cultivation of State Forestry Administration, Research Institute of Forestry, Chinese Academy of Forestry, Beijing, China

**Keywords:** litter leaf, mixed decomposition, microbial community, *Populus* × *canadensis* Moench, *Pinus sylvestris* var. *mongolica*

## Abstract

Decomposition of forest litter is an essential process for returning nutrients to the soil, which is crucial for preserving soil fertility and fostering the regular biological cycle and nutrient balance of the forest ecosystem. About 70% of the land-based forest litter is made up primarily of leaf litter. However, research on the complex effects and key determinants of leaf litter decomposition is still lacking. In this study, we examined the characteristics of nutrient release and microbial diversity structure during the decomposition of three types of litter in arid and semi-arid regions using 16S rRNA and ITS sequencing technology as well as nutrient content determination. It was revealed that the nutrient content and rate of decomposition of mixed litters were significantly different from those of single species. Following litter mixing, the richness and diversity of the microbial community on leaves significantly increased. It was determined that there was a significant correlation between bacterial diversity and content (Total N, Total P, N/P, and C/P). This study provided a theoretical framework for investigating the decomposition mechanism of mixed litters by revealing the microbial mechanism of mixed decomposition of litters from the microbial community and nutrient levels.

## Introduction

Litter, also referred to as organic debris, is a general term for all organic matter in the ecosystem created by the withering of above-ground plant components and returned to the surface as a source of material and energy for decomposers to maintain ecosystem functions (Schlesinger and Lichter, 2001). Leaf litter makes up about 70% of the above-ground forest litter among them. Leaf litter currently dominates 72% of the research on litter in forest ecosystems (Jia B. R. et al., 2018; Wymore et al., 2018; Nicolas et al., 2019). The primary source of organic matter and nutrients in forest soils is litter decomposition, which also plays a crucial role in the energy flow and nutrient cycling in forest ecosystems (Swift et al., 1979). It also acts as a link between the two carbon pools found in soil and living things (Manzoni et al., 2010). It is the catalyst for biogeochemical cycles and significantly affects climate change (Nevins et al., 2018). The decomposition of leaf litter is a crucial indicator of the functioning of an ecosystem’s health because it can return plant carbon, nitrogen and phosphorus to the soil and the atmosphere, as well as other nutrients. This can have an impact on the global element balance, particularly the carbon balance (Cotrufo et al., 2015). Statistics show that every year, 68 GtC of carbon dioxide is released into the atmosphere by the decomposition of litter, making up roughly 70% of the annual carbon flux worldwide (Raich and Schlesinger, 1992). It also serves as a rich source of nutrients for microorganisms, and is crucial for maintaining soil fertility, promoting the regular biological cycle, and maintaining the nutrient balance in forest ecosystems (Manzoni et al., 2008; Jiang et al., 2013), all of which have long been of great concerned to researchers (Gui et al., 2017; Tan et al., 2020).

The nature of the litter, the composition and diversity of the decomposer community, soil conditions, and the climate all have an impact on how quickly litter decomposes (Handa et al., 2014; Stefano, 2017; Han et al., 2019; Sébastien et al., 2021). Climate has a significant impact on the rate at which litter decomposes both globally and locally. On a smaller scale, the type of the litter itself has the greatest impact on how quickly it decomposes. Litter leaf quality largely determines the decomposition of litter and the release of nutrients and minerals to the soil (Lucas-Borja et al., 2018), especially the carbon-nitrogen ratio of the litter (Bradford et al., 2016). The quality of this litter substrate will reduce its decomposition rate (Xiao et al., 2019), and higher C/N and C/P will inhibit the decomposition of leaf litter and its release of nutrients (Lucas-Borja et al., 2018). Coniferous litter typically has a higher C/N than broad-leaved litter. In view of this, understanding the nutrient cycle of leaf litter depends on understanding the stoichiometric characteristics of different shelterbelt species’ leaf litter.

The qualities of leaf litter (N and C/N) vary greatly between different plants, and the properties of leaf litter and the chemical structure of organic carbon are significant factors affecting the decomposition of the litter (Cotrufo et al., 2015). The primary source of leaf litter decomposition and the primary force driving the decomposition occurs when plant leaves fall to the ground (Davidson and Janssens, 2006; Cornelissen et al., 2007). The quality of the litter and its specialized microorganisms work together to cause litter to decompose. Uncertainty persists regarding the microbial community changes that occur as various plant leaf litter decomposes.

Leaf microbes are microorganisms that live as epiphytes or parasites on the leaves of plants (Jia T. et al., 2018; Yao et al., 2019). Phyllosphere microbial communities have different makeups depending on the type of plant and the environment it lives in. Although different species’ phyllosphere microbial communities differ significantly from one another (Bao et al., 2019), they all share a similar structure (Wallace et al., 2018). As plants progressively wither, variability of phyllosphere microbes gradually increases (Ferreira et al., 2016), and changes in phyllosphere microbial communities during decomposition (Whipps et al., 2010; Kembel et al., 2014) are influenced by leaves’ strong influence on physicochemical properties. The abundance of microbial communities significantly increased along with the degree of physical and biological fragmentation of litter (Guerreiro et al., 2017). Fungi are thought to play a significant role in the biogeochemical cycles and the decomposition of leaf litter (Osono et al., 2004). Saprophytic basidiomycetes are the primary decomposers of litter and have the ability to break down materials that are difficult to decompose, like the cellulose found in woody litter (Eichlerová et al., 2015; Urbanová et al., 2015; Baldrian, 2017). Bacteria, which can break down cellulose and hemicellulose, are also necessary for the decomposition of leaf litter. It is not yet clear, though, whether the succession of fungi during the decomposition of litter is consistent with bacterial succession. Additionally, and There is no information on the dynamic changes in phyllosphere microbial composition over time involving multiple decomposition stages, and most studies only concentrate on one stage of plant leaf decomposition or associated leaf litter surface microbes. A though understanding of the connection between the microbial community and litter decomposition over time can be gained by studying the phyllosphere’s microbial community.

Early research concentrated on the decomposition of a single leaf litter, but the majority of leaf litter in forest ecosystems was mixed, which had an impact on the nutrient release law during the decomposition process (Lu et al., 2020). Coniferous and broad-leaved litter’s physicochemical differences can have an impact on the structure and operation of the litter microbial community (Lin et al., 2021). In general, mixed litter has higher fungal and bacterial abundances and microbial community diversity than single litter, and they have significantly different microbial community compositions (Sun et al., 2018; Pereira et al., 2019; Zhang et al., 2019). This could cause variations in the metabolic functions of microbial communities (Li et al., 2015).

Microorganisms are the primary actors in the decomposition of litter, according to previous research (Muscolo et al., 2014; Lin et al., 2015; Scheibe et al., 2015), and they decompose more quickly in the decomposition of mixed litter than in the decomposition of asingle litter (Patoine et al., 2017; Tonin et al., 2017). According to some studies, the structure and operation of microbial communities can be affected in specific ways by the mixing of litter (Wang et al., 2019). This is due to the mixed litter’s higher levels of carbon sources and nutrients, which increase the nutrient supply to microorganisms and accelerate decomposition through complementary effects (Cline and Zak, 2015; Xiao et al., 2019). Evidently, there is currently a dearth of knowledge regarding the impact of microbial composition on litter decomposition. In addition, research on the structure of the microbial community involved in the decomposition of litter focuses less on that process and instead primarily on the structure of the microbial community in the soil (Sun et al., 2017). Therefore, it is useful to understand the mechanism of litter decomposition to study the changes in microbial community composition during decomposition.

“Three-North” Shelter Forest Program was launched by the Chinese government in 1978 in Northwest China, North China and Northeast China (collectively referred to as the “Three-North” Regions) in an effort to improve the ecological environment. Important tree species for afforestation in the “Three-North” Shelter Forest Program include *Populus* × *canadensis* Moench and *Pinus sylvestris* var. *mongolica*, both of which have straight trunks, a love of light, and a resistance to cold. In order to study the mixed decomposition characteristics of litter, *Populus* × *canadensis* Moench and *Pinus sylvestris* var. *mongolica* plantation litter in arid and semi-arid regions was chosen as the research object. The following hypotheses were put forth: (1) mixed litter decomposition altered the physicochemical characteristics of leaves in comparison to pure *Pinus sylvestris* var. *mongolica* forests; (2) conifer litter’s microbial community structure and diversity were affected by the addition of *Populus × canadensis* Moench litter; (3) the decomposition of *Pinus sylvestris* var. *mongolica* litter is accelerated by a mixture of withered leaves from coniferous and broad-leaved species. To provide a theoretical foundation for the decomposition of leaf litter, it’s necessary to reveal the interactions in mixed decomposition of *Populus × canadensis* Moench and *Pinus sylvestris* var. *mongolica*.

## Materials and methods

### Site description

The study site was established in the Liaohe Plain Forest Ecological Station of the National Forestry and Grassland Administration which is Fujia Forest Farm in Changtu County, Tieling City, Liaoning Province (43°21′143″–42°53′623″N, 123°53′623″–123°53′623″E). It is located in the southeastern part of Horqin Sandy Land, at the junction of Liaoning Province, Jilin Province, and Inner Mongolia Autonomous Region. The topography is flat, and there are a small number of sand dunes in the Liaohe throttle. It is a temperate semi-humid and semi-arid continental climate with an average annual precipitation of 400–550 mm, mainly concentrated in July and August, and evaporates 1,843 mm per year. The maximum temperature was 35.6°C, the lowest temperature was −31.5°C, and the average daily temperature was 6.3°C. The soil type contains low levels of organic substances and other nutrients.

### Litter sampling

A litter collector of 1 m *×* 1 m was set up at the study site, and fresh litter samples of *Populus × canadensis* Moench and *Pinus sylvestris* var. *mongolica* were collected in October 2020. Litter samples were then mixed and stored in ice boxes for immediate shipment back to the lab. The leaves of *Populus × canadensis* Moench and *Pinus sylvestris* var. *mongolica* were air-dried and put into drop bags. The collected litter was packed into a nylon mesh decomposition bag with a size of 20 cm × 20 cm (the aperture was 1 mm × 1 mm, i.e., 16 mesh). The total weight of each bag was 8 g, and the mass ratios of litter leaves of *Populus × canadensis* Moench and *Pinus sylvestris* var. *mongolica* were 1:0, 1:1, and 0:1 (YS, YZ, and ZS in subsequent analysis). The litter bags were kept in a dehumidified, air-conditioned room. A decomposition test was set up under a *Pinus sylvestris* var. *mongolica* stand in April 2021. Eight litter decomposition bags of different proportions were set in each plot, and a total of four plots were repeated. In July 2021, bags’ surface waste was carefully removed, the litter bag retrieved and stored in an ice box for immediate transport back to the laboratory. Then 8 litter bags from the same plot were mixed, weighed and divided into 2 parts. A piece of the litter was dried at 65°C to a constant weight, then weighed to calculate its moisture content and total dry weight. The dried litter samples were crushed and pulverized through a 0.15 mm sieve (100 mesh). The chemical properties of dried litter samples were determined after crushing, including total carbon (total C), total nitrogen (total N), and total phosphorus (total P). The other part was put into a sterilization bag and stored in a − 80°C refrigerator for the determination of microbial diversity.

### Determination of litter characteristics

An elemental analyzer (Elementar Vario EL III, Germany) was used to measure total C and total N in litter (Song et al., 2015). The total P in litter was determined by the molybdenum-antimony anti-spectrophotometric method (Ba et al., 2020). The weight loss rate of litter (Dw) was determined by the drying method.


$$\mathrm{Dw}=\frac{\mathrm{Mo}-\mathrm{Mt}}{\mathrm{Mo}}\times 100\%$$

In the formula: Dw is the weight loss rate of litter (%), Mo is the dry weight (g) of the sample in the decomposition bag at the time of delivery, and Mt. is dry weight (g) of litter after sample decomposition.

### DNA extraction and amplification sequencing

Total DNA extraction was carried out according to the MoBio PowerSoil DNA Isolation Kit (MP Biomedicals, Santa Ana, CA, United States) Kit procedure of OMEGA, and each sample was weighed at about 0.5 g. A NanoDrop ND-1000 spectrophotometer (Thermo Fisher Scientific, Waltham, MA, United States) determined the quantity and quality of the extracted DNA. Primers 338F (5′-ACTCCTACGGGAGGCAGCA-3′) and 806R (5′-GGACTACHVGGGTWTCTAAT-3′) were used to amplify the V3-V4 region of the bacterial 16S rRNA gene (Claesson et al., 2009). The fungal ITS region was amplified with primers ITS5 (5′-GGAAGTAAAAGTCGTAACAAGG-3′) and ITS2 (5′-GCTGCGTTCTTCATCGATGC-3′; White, 1994). The PCR amplification system has a total of 25 μl, including: DNA template 2 μl, each 1 μl upstream and downstream primers (10 μmol·L^−1^), buffer 5 μl, Q5 high-fidelity buffer 5 μl, 0.25 μl high-fidelity DNA polymerase (5 U·μl^−1^), dNTP (2.5 mmol·L^−1^) 2 μl, ultrapure water (dd H_2_O) 8.75 μl. PCR amplification conditions were first pre-denatured at 98°C for 2 min, then repeated 25 times in a cycle of 98°C for 15 s, 55°C for 30 s, and 72°C for 30 s, and finally extended at 72°C for 5 min. The PCR amplicons were purified by using Agincourt AM-Pure Beads (Beckman Coulter, Indianapolis, IN), and quantified by using the Pico Green dsDNA detection kit (Invitrogen, Carlsbad, CA, United States). The PCR products were sequenced by using the Illumina NovaSeq 6000 sequencing platform by Shanghai Personal Biotechnology Co., Ltd.

### Microbial bioinformatics analysis

QIIME2 (2019.4) and UPARSE Pipeline were used to carry out correlation calculation and analysis on the original data sequences of high-throughput sequencing, and the sequence length was screened (Caporaso et al., 2010). QIIME2’s UCLUST, a sequence alignment tool, was used to merge the effective sequences with more than 97% similarity to OTU, and the sequence with the highest abundance of each OTU was selected as the representative sequence of OTU (Edgar, 2010), and compared with the template sequence of Silva database (Release123)^1^ to obtain the classification information of each OTU (Quast et al., 2012). Then, according to the number of sequences contained in each sample, a matrix file of OTU abundance in each sample is constructed, namely, OTU table. The richness indices of OTUs and diversity index, including Shannon index, Simpson index, and Chao1 index, were analyzed.

### Statistical analysis

For data processing, Microsoft Excel (2019) was used, and the data in the table were all repeated Average ± Standard Error. SPSS 26.0 was used for statistical analysis. One-way analysis of variance was used to analyze the differences in physicochemical properties of different litter leaves, and two-way correlation analysis was used to compare the relationship between physicochemical properties of litter leaves and microbial diversity. Ecologists use alpha diversity and beta diversity indices to characterize the diversity of species within and between habitats, respectively, to comprehensively evaluate their overall diversity (Whittaker, 1960; Whittaker, 1972). In order to comprehensively assess the alpha diversity of microbial communities, Chao1 (Chao, 1984) and Observed species indices were used to characterize richness, and Shannon (Chiani, 1948; Shannon, 1948) and Simpson (Simpson, 1949) indices were used to characterize diversity. Evenness was characterized by Pielou’s evenness index (Pielou, 1966), and coverage by Good’s coverage index (Good, 1953). And the ggplot2 package of R (R v.3.4.4) was used to draw a boxplot. Principal coordinates analysis (PCoA) is one of the most classic unconstrained sorting (Classical Multidimensional Scaling, cMDScale) analysis methods (Alban, 2010). The differences in the β-diversity of litter leaves were analyzed and compared according to the OTU table and the ape package of R (R v.3.4.4). Among the samples, shared and unique OTUs of litter microbial communities were used in R (R v.3.4.4) and the “Venn Diagram” package to create Venn diagrams. Heatmaps for the top 50 taxonomic genera in each sample were constructed using R (R v.3.4.4) and the pheatmap package. The linear discriminant analysis (LDA) effect size (LEfSe) method was used to detect potentially biomarker-rich taxa based on a cross-group normalized relative abundance matrix using default parameters. The CoHeatmap analysis was carried out by the genescloud tool^1^ utilizing the Spearman rank correlation coefficient algorithm, *R* = 0.5. The matrix was constructed using Galaxy, an online interactive analysis of microbial community data.

## Results

### Characterization of physicochemical properties of different species of litter

There were extremely significant differences in Dw (*p* < 0.01, *F* = 3296.941), total C (*p* < 0.01, *F* = 20.538), total P (*p* < 0.05, *F* = 7.668), C/N (*p* < 0.01, *F* = 68.247), N/P (*p* < 0.01, *F* = 21.415), and C/P (*p* < 0.01, *F* = 26.931) among the three types of leaf litter, but there was no significant difference in the content of total N (*p* > 0.05, *F* = 4.355), as shown in Table 1. The total C content of YS was 679.25 g·kg^−1^, which was significantly lower than that of YZ and ZS, while the total P content (2.31 g·kg^−1^) was significantly higher than that of YZ and ZS (*p* < 0.01). The total C, total N and total P contents of YZ were 879.00 g·kg^−1^, 57.75 g·kg^−1^ and 1.69 g·kg^−1^, respectively, which were higher than those of ZS. The significance rules of N/P and C/P of the three types of litter were consistent, and ZS was extremely significantly higher than YS and YZ (*p* < 0.01). The C/N of YZ was significantly different from that of YS (*p* < 0.01), but not significantly different from that of ZS (*p* > 0.05). Dw intuitively represents the rate at which litter decomposes. As can be seen from Table 1, YS had the highest decomposition rate, followed by YZ, and ZS was the lowest.

**Table 1:** Differences in physicochemical properties of three types of litter.

Different samples	Total C/g·kg^−1^	Total N/g·kg^−1^	Total P/g·kg^−1^	C/N	N/P	C/P	Dw/%
YS	679.25 ± 59.28B	54.00 ± 3.16A	2.31 ± 0.85A	12.56 ± 0.36B	25.21 ± 7.06B	315.89 ± 86.75B	20.91 ± 0.09A
ZS	824.50 ± 12.87A	54.00 ± 0.82A	0.78 ± 0.15B	15.27 ± 0.10A	71.75 ± 14.37A	1094.78 ± 214.60A	17.44 ± 0.05C
YZ	879.00 ± 50.50A	57.75 ± 1.50A	1.69 ± 0.43AB	15.21 ± 0.53A	35.79 ± 8.78B	544.44 ± 133.58B	20.20 ± 0.02B
F test	20.538	4.355	7.668	68.247	21.415	26.931	3296.941

Data were means ± standard error (*n* = 4). Degrees of freedom = 11. Different capital letters meant significant difference at 0.01 level. YS: *Populus* × *canadensis Moench*; YZ: *Populus* × *canadensis Moench* – *Pinus sylvestris var. mongolica*; ZS: *Pinus sylvestris var. mongolica*.

### Characteristics of microbial community composition In different types of litter leaves

**Figure 1:**
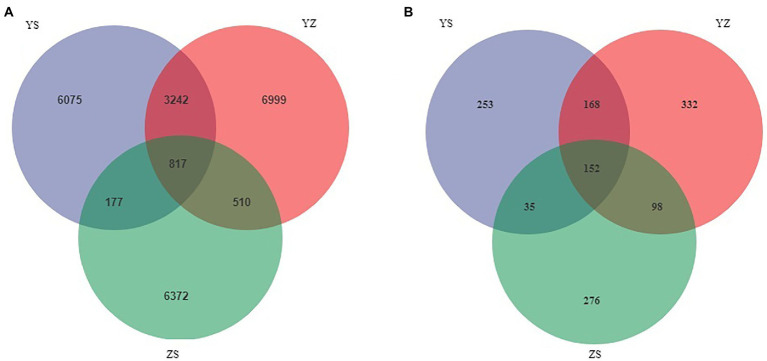
Venn diagrams showed the unique and shared OTU of litter microorganisms from three different samples. **(A)** unique and shared OTUs of leaf litter bacteria in three different samples; **(B)** unique and shared OTUs of leaf litter fungal in three different samples. YS: *Populus* × *canadensis* Moench; YZ: *Populus* × *canadensis* Moench-*Pinus sylvestris* var. *mongolica*; ZS: *Pinus sylvestris* var. *mongolica*.

**Figure 2:**
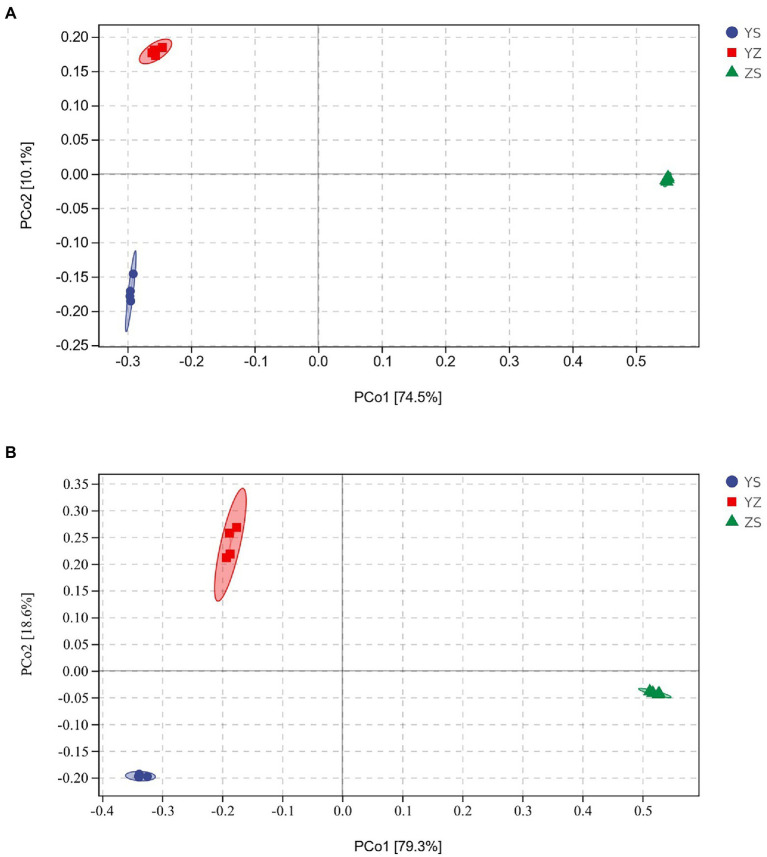
Priniciple coordinate analysis (PCoA) of Bray-Curtis’ diatance from all samples. **(A)** PCoA of bacteria communities in different samples; **(B)** PCoA of fungal communities in different samples. YS: *Populus × canadensis* Moench; YZ: *Populus × canadensis* Moench-*Pinus sylvestris* var. *mongolica*; ZS: *Pinus sylvestris* var. *mongolica*.

Venn plots were used to compare OTUs common to and unique to bacterial and fungal communities across all samples. At the bacterial level, a total of 24,192 OTUs were detected. Among them, the OTUs of YS, YZ and ZS were 10,311, 11,586, and 7,876, respectively. The total OTU of the three samples was 817. The OTUs specific to YS, YZ and ZS were 6,075, 6,999, and 6,372, respectively (Figure 1A). At the fungal level, however, the number of detected OTUs was only 1,314. Among them, the OTUs of YS, YZ and ZS are 608, 750, and 561. The common OTU among them was 152. The OTUs specific to YS, YZ, and ZS were 253, 332, and 276, respectively (Figure 1B). Principal coordinates analysis (PCoA) based on Bray-Curtis distance analysis of the different samples showed that the composition of bacterial and fungal communities between YS, YZ, and ZS were all different, forming three disjoint confidence ellipses, and all split along the PCo1 axis (Figure 2).

**Figure 3:**
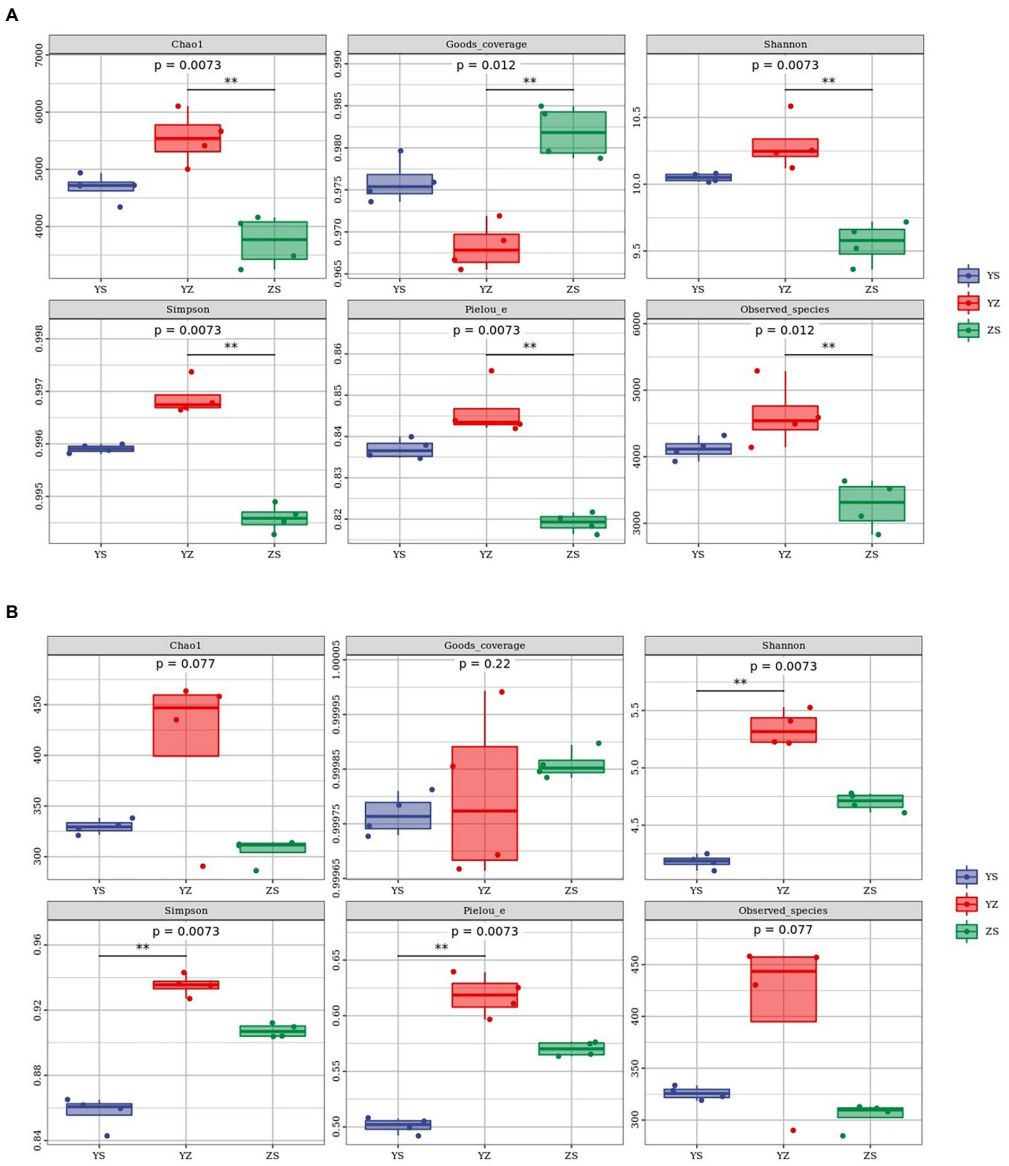
Litter microbial diversity index in YS, YZ and ZS. **(A)** Alpha diversity analysis of leaf litter bacterial community. **(B)** Alpha diversity analysis of leaf litter fungal community. YS: *Populus × canadensis* Moench; YZ: *Populus × canadensis* Moench-*Pinus sylvestris* var. *mongolica*; ZS: *Pinus sylvestris* var. mongolica. ** meant significant difference at 0.01 level.

Alpha diversity refers to an index in ecology to estimate the richness, diversity and evenness of species and flora. Regarding the bacterial alpha-diversity, there was a distinction between the alpha diversity of the three litter types, including Chao1 index (*F* = 20.99; *p* = 0.0073), Goods_coverage (*F* = 22.77; *p* = 0.012), Shannon index (*F* = 25.98; *p* = 0.0073), Simpson index (*F* = 83.11; *p* = 0.0073), Pielou_e index (*F* = 43.12; *p* = 0.0073) and Observed_species (*F* = 14.18; *p* = 0.012). The six indexes of YZ were significantly different from those of ZS (*p* < 0.01), but the indexes of YZ and YS were not significantly different (*p* > 0.05). From Figure 3A, it could be seen intuitively that the Chao1 index, Pielou_e index, Shannon index, Simpson index and Observed_species index of YZ are the highest, followed by YS and ZS the lowest. And the Goods_coverage index showed the opposite law, namely ZS > YS > YZ. At the fungal level, only YS and YZ had significant differences between Pielou_e index (*F* = 97.84; *p* = 0.0073), Shannon index (*F* = 126.27; *p* = 0.0073), and Simpson index (*F* = 118.38; *p* = 0.0073), and there were no significant differences between the other indicators and samples (*p* > 0.05). The same as bacteria, YZ had the highest Chao1 index, Pielou_e index, Shannon index, Simpson index, and Observed_species index. In the above index, except that the Chao1 index of YS was higher than ZS, the rest were ZS higher than YS (Figure 3B).

**Figure 4:**
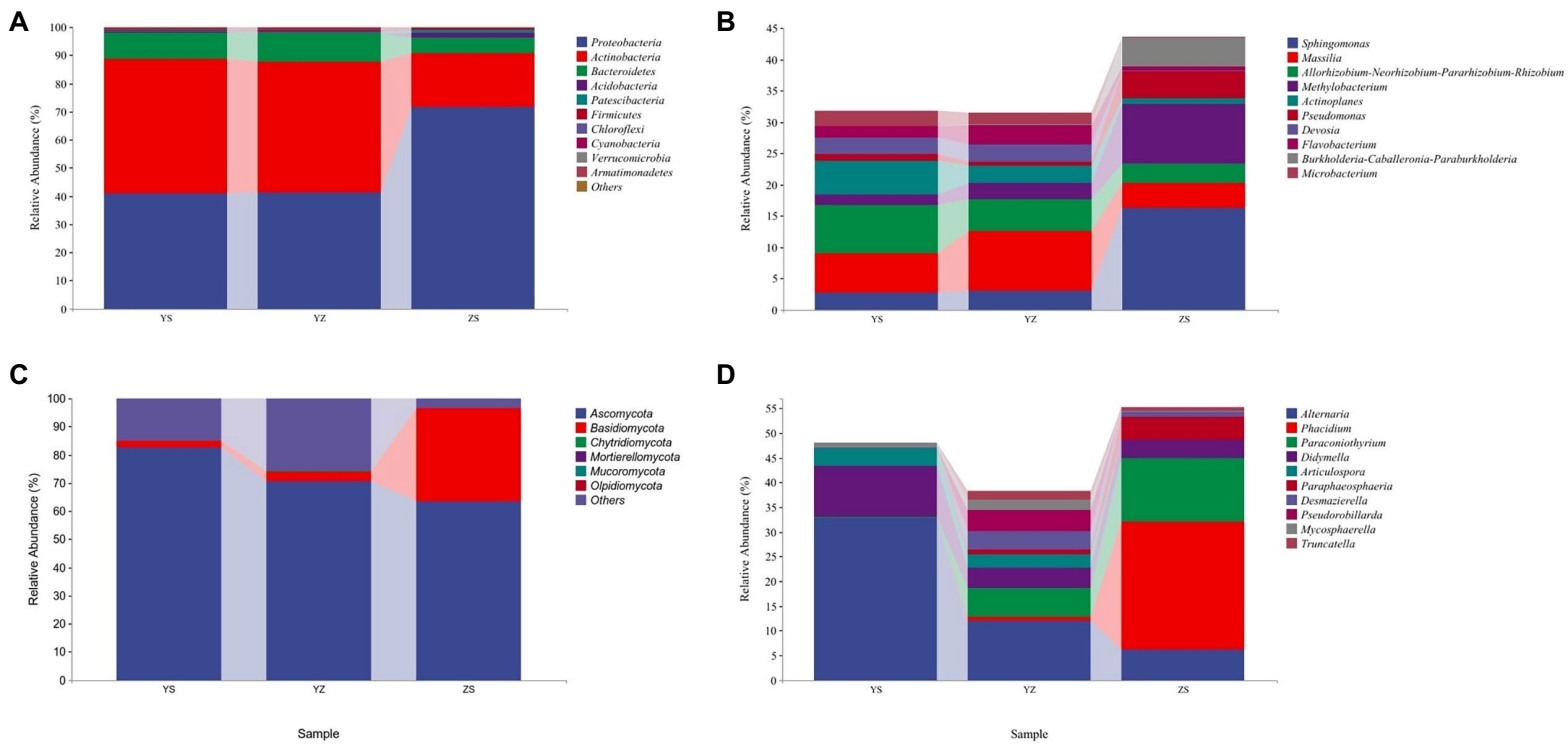
The relative abundance of microorganisms at pyhlum and genus levels in litter from different samples. **(A)** The relative abundance of dominant bacterial community at the phylum level; **(B)** The relative abundance of dominant bacterial community at the genus level (not showing others); **(C)** The relative abundance of dominant fungal community at the phylum level; **(D)** The relative abundance of dominant fungal community at the genus level (not showing others). YS: *Populus × canadensis* Moench; YZ: *Populus × canadensis* Moench-*Pinus sylvestris* var. *mongolica*; ZS: *Pinus sylvestris* var. *mongolica.*

The relative abundance of microorganisms in the samples of the three litter types was counted, and the top 10 relative abundances were drawn at the phylum level (others are shown) and genus level (others not shown), respectively, as shown in Figure 4. At the bacterial phylum level, the top 10 relative abundances were Proteobacteria, Actinobacteria, Bacteroidetes, Acidobacteria, Patescibacteria, Firmicutes, Chloroflexi, Cyanobacteria, Verrucomicrobia, Armatimonadetes. The relative abundance of Proteobacteria was the highest in ZS at 71.65%, followed by YZ (41.32%) and YS (41.00%). On the contrary, Actinobacteria had the highest relative abundance in YS at 47.6%, followed by YZ (46.23%) and ZS (19.05%). The relative abundance of Bacteroidetes was YZ > YS > ZS (Figure 4A). At the fungal phylum level, Ascomycota, Basidiomycota, Chytridiomycota, Mortierellomycota, Mucoromycota and Olpidiomycota were relatively abundant. Among them, only Ascomycota and Basidiomycota have relative abundance higher than 1%. The relative abundance of Ascomycota is the highest in YS at 82.75%, followed by YZ (70.70%) and the lowest in ZS (63.39%); the relative abundance of Basidiomycota is just the opposite, that is, ZS (33.29%) > YZ (3.38%) > YS (2.08%; Figure 4C).

**Figure 5:**
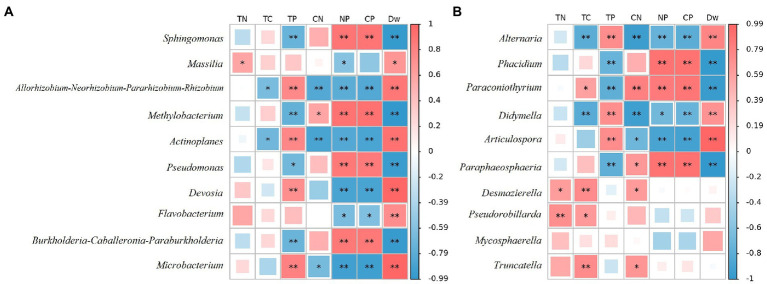
CoHeatmap shows the 10 genera relative abundance and chemical composition in various samples of litter. **(A)** Bacteria; **(B)** Fungi. The legend shows the correlation coefficient values, with red representing positive correlations and blue representing negative correlations. Shades of color indicate the strength of the correlation. *represents *p* < 0.05; **represents *p* < 0.01. TN: Total N; TC: Total C; TP: Total P; CN: C/N; NP: N/P; CP: C/P.

At the bacterial genus level, the top 10 relative abundances were *Sphingomonas*, *Massilia*, *Allorhizobium-Neorhizobium-Pararhizobium-Rhizobium*, *Methylobacterium*, *Actinoplanes*, *Pseudomonas*, *Devosia*, *Flavobacterium*, *Burkholderia-Caballeronia-Paraburkholderia*, and *Microbacterium* (Figure 4B). It is evident from Figure 5A that there was a correlation between the physicochemical characteristics of the litter and these 10 genera. *Sphingomonas*, *Methylobacterium*, *Pseudomonas*, *Burkholderia-Caballeronia-Paraburkholderia* were significantly positively correlated with litter N/P, C/P (*p* < 0.01), and negatively correlated with Dw (*p* < 0.01). *Allorhizobium-Neorhizobium-Parararhizobium-Rhizobium*, *Actinoplanes*, *Devosia*, *Flavobacterium*, and *Microbacterium* were significantly negatively correlated with litter N/P, C/P (*Flavobacterium*, *p* < 0.05; other else, *p* < 0.01) was significantly negatively correlated with N/P and positively correlated with TN, Dw (*p* < 0.05).

At the fungal genus level, the top 10 relative abundances were *Alternaria*, *Phacidium*, *Paraconiothyrium*, *Didymella*, *Articulospora*, *Paraphaeosphaeria*, *Desmazierella*, *Pseudorobillarda*, *Mycosphaerella*, and *Truncatella* (Figure 4D). Similarly, as can be seen from Figure 5B, *Alternaria*, *Didymella*, and *Articulospora* were significantly negatively correlated with litter N/P, C/P (Didymella, *p* < 0.05; other else, *p* < 0.01), and positively correlated with Dw (*p* < 0.01). *Phacidium*, *Paraconiothyrium*, and *Paraphaeosphaeria* were significantly positively correlated with litter N/P, C/P (*p* < 0.01), and negatively correlated with Dw (*p* < 0.01). Nevertheless, the relatively low levels of *Desmazierella*, *Pseudorobillarda*, *Mycosphaerella*, and *Truncatella* was not significantly correlated with Dw (*p* > 0.05).

**Figure 6:**
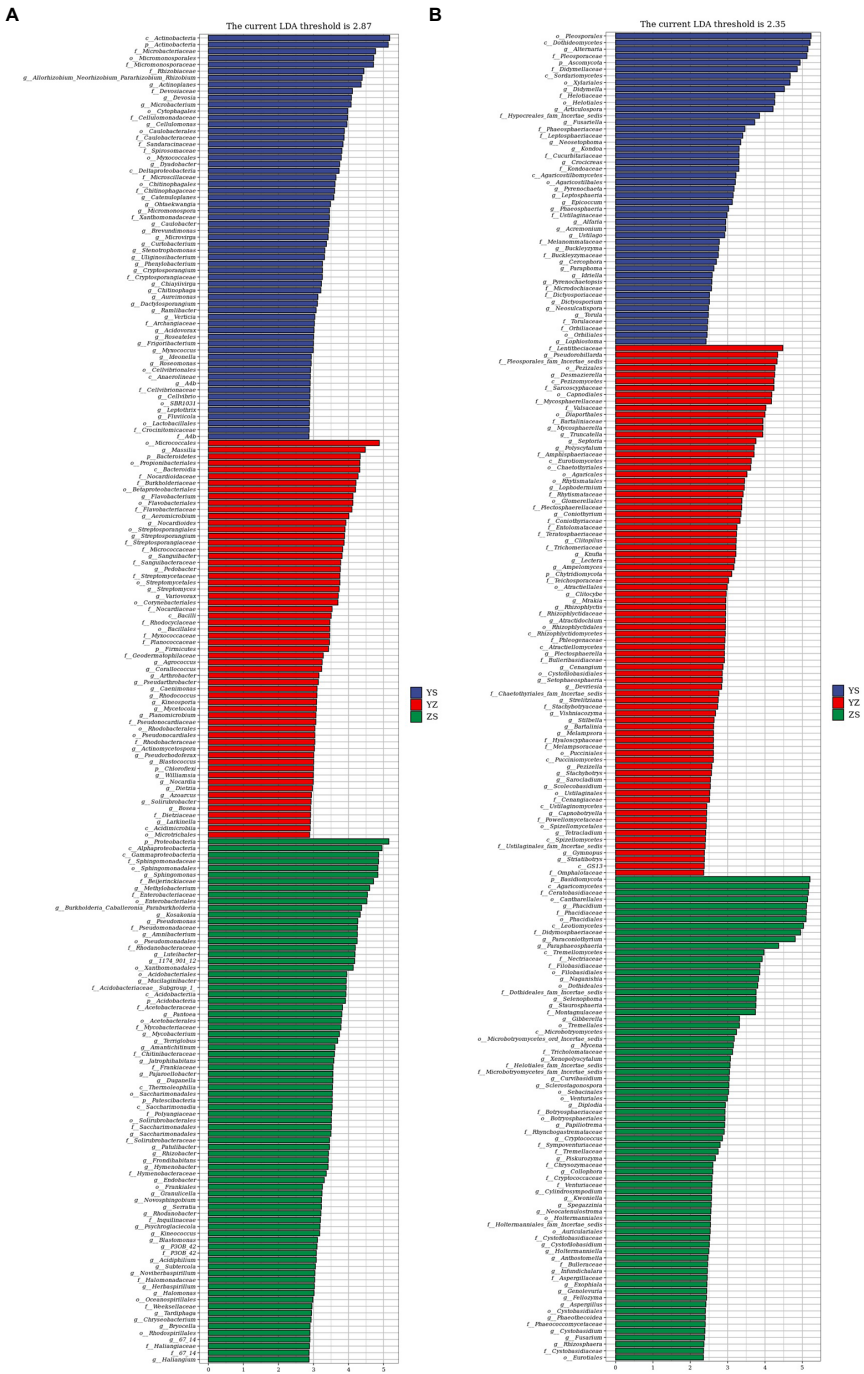
Microbial community of different leaf litter ratios with significantly different taxa. **(A)** litter bacterial communities; **(B)** litter fungal communities. YS: *Populus × canadensis* Moench; YZ: *Populus × canadensis* Moench-*Pinus sylvestris* var. *mongolica*; ZS: *Pinus sylvestris* var. *mongolica.* The longer the length, the more significant the difference between the taxon units, and the different color of the bar chart indicates the higher abundance sample group corresponding to the taxon.

**Figure 7:**
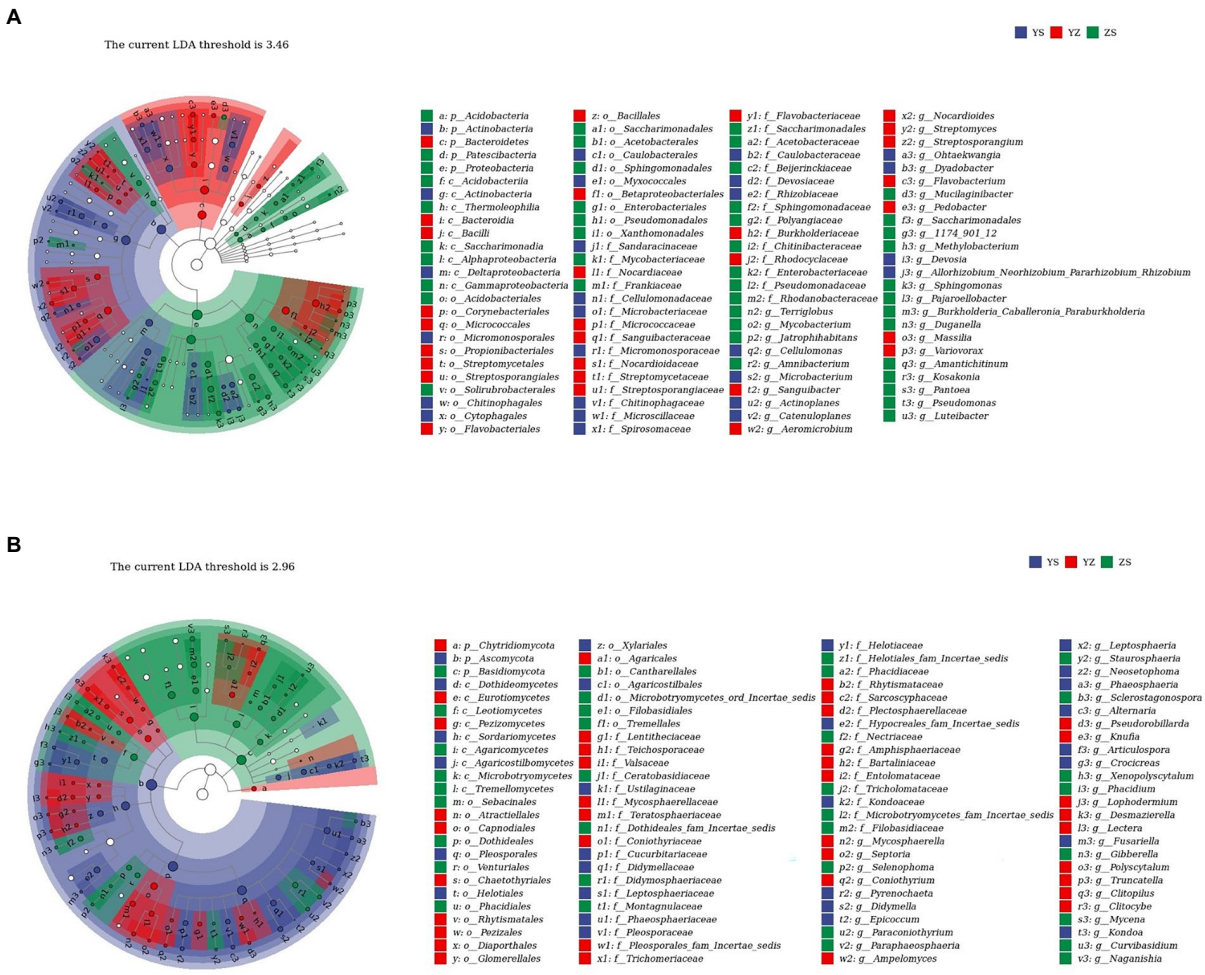
**(A)** Lefse with an LDA of 3.46 indicates that the 100 groups are significantly different between litter bacterial communities of YS, YZ, and ZS. **(B)** Lefse with an LDA of 2.96 indicates that the 100 groups are significantly different between litter fungal communities of YS, YZ, and ZS. YS: *Populus × canadensis* Moench; YZ: *Populus × canadensis* Moench-*Pinus sylvestris* var. *mongolica*; ZS: *Pinus sylvestris* var. *mongolica.* Circle radiation from inner to outer of evolutionary branch figure represents the classification of the level from phylum door genus; every small circle represents the level of a classification in different classification level. The diameter of the circle is prportional to the relative abundance.

Lefse is a recent analysis method based on Linear discriminant analysis (LDA) effect size. Its essence is to combine linear discriminant analysis with nonparametric Kruskal-Wallis and Wilcoxon rank sum tests to screen for key biomarkers (i.e., key community members; Segata et al., 2011). With LDA effect size scores >4.5, 16 bacterial taxa were significantly different across treatments (Figure 6A). When LDA effect size scores were > 5, 3 bacterial taxa were significantly different in litter from ZS and YS. Among them, at the bacterial phylum level, the main enriched bacterial taxa in YS leaf litter were Actinobacteria, ZS was mainly enriched by Proteobacteria, and YZ was Bacteroidetes (Figure 7A). As shown in Figure 6B, when the LDA effect size score was >4, the relative abundances of 19 fungal taxa were significantly different among different treatments (*p* < 0.05). Regarding the fungal alpha-diversity, Ascomycota was mainly enriched in YS litter, while Basidiomycota was mainly enriched in ZS (Figure 7B).

The Bray-Curtis-based heatmap showed that the litter bacterial communities of YS and YZ were clustered together, indicating that the litter leaf communities from ZS were clearly distinct from those of YS and YZ (Supplementary Figure S1). The fungal community of the litter also showed the same conclusion (Supplementary Figure S2).

### Correlation between physicochemical properties of litter and microbial community diversity in litter

Regarding the bacterial alpha-diversity, total N of leaf litter was significantly positively correlated with Pielou_e (*r* = 0.595, *p* < 0.05) and Simpson index (*r* = 0.588, *p* < 0.05); total P was significantly positively correlated with Pielou_e (*r* = 0.642, *p* < 0.05) and Shannon index (*r* = 0.587, *p* < 0.05); N/P was significantly positively correlated with Goods_coverage (*r* = 0.581, *p* < 0.05); N/P and C/P were significantly negatively correlated with Chao 1 index (*r* = −0.651, *p* < 0.05; *r* = −0.626, *p* < 0.05), Observed_species index (*r* = −0.704, *p* < 0.05; *r* = −0.683, *p* < 0.05), Pielou_e index (*r* = −0.760, *p* < 0.01; *r* = −0.735, *p* < 0.01), Shannon index (*r* = −0.751, *p* < 0.01; *r* = −0.730, *p* < 0.01) and Simpson index (*r* = −0.729, *p* < 0.01; *r* = −0.699, *p* < 0.05). Dw was significantly positively correlated with Chao 1 index (*r* = 0.693, *p* < 0.05), Goods_coverage (*r* = 0.623, *p* < 0.05), Pielou_e index (*r* = 0.728, *p* < 0.01), Shannon index (*r* = 0.819, *p* < 0.01) and Simpson index (*r* = 0.794, *p* < 0.01). Dw was significantly negatively correlated with Observed_species index (*r* = −0.632, *p* < 0.05). There was no significant relationship between litter total C and bacterial community diversity index (Table 2).

**Table 2:** Correlation between litter physicochemical properties and bacterial community diversity.

	Chao1	Goods_coverage	Observed_species	Pielou_e	Shannon	Simpson
Total N	0.423	−0.404	0.406	0.595^*^	0.478	0.588^*^
Total C	0.124	−0.180	0.046	0.113	0.053	0.168
Total P	0.421	−0.314	0.515	0.642^*^	0.587^*^	0.567
C/N	−0.074	−0.007	−0.165	−0.172	−0.192	−0.098
N/P	−0.651^*^	0.581^*^	−0.704^*^	−0.760^**^	−0.751^**^	−0.729^**^
C/P	−0.626^*^	0.553	−0.683^*^	−0.735^**^	−0.730^**^	−0.699^*^
Dw	0.693^*^	0.623^*^	−0.632^*^	0.728^**^	0.819^**^	0.794^**^

*n* = 12, ** means significant correlation at *p* < 0.01level; * means significant correlation at *p* < 0.05 level.

Regarding the fungal alpha-diversity, total N of leaf litter was significantly positively correlated with Shannon index (*r* = 0.602, *p* < 0.05); total C and C/N were significantly positively correlated with Pielou_e index (*r* = 0.841, *p* < 0.01; *r* = 0.835, *p* < 0.05), Shannon index (*r* = 0.846, *p* < 0.01; *r* = 0.791, *p* < 0.01) and Simpson index (*r* = 0.888, *p* < 0.01; *r* = 0.886, *p* < 0.01). There was no significant relationship between total P, N/P, C/P, Dw and microbial diversity index of litter (Table 3).

**Table 3:** Correlation between litter physicochemical properties and fungal community diversity.

	Chao1	Goods_coverage	Observed_species	Pielou_e	Shannon	Simpson
Total N	0.563	−0.168	0.567	0.517	0.602^*^	0.538
Total C	0.461	0.068	0.472	0.841^**^	0.846^**^	0.888^**^
Total P	0.143	−0.407	0.130	−0.417	−0.313	−0.454
C/N	0.293	0.204	0.307	0.835^**^	0.791^**^	0.886^**^
N/P	−0.280	0.446	−0.267	0.282	0.159	0.343
C/P	−0.236	0.435	−0.222	0.344	0.224	0.406
Dw	0.378	−0.414	0.368	−0.288	−0.134	−0.345

*n* = 12, ** means significant correlation at *p* < 0.01level; * means significant correlation at *p* < 0.05 level.

## Discussions

“Litter mass,” according to Swift et al., (1989), refers to the physicochemical composition of litter. It is the nutrient composition and structure, which includes easily degradable parts (N, P) and resistant organic parts (lignin, cellulose, etc.). The primary controlling factor influencing the rate of litter decomposition is thought to be litter quality (Cornwell et al., 2008). The three elements C, N, and P are currently the primary physicochemical elements involved in research on litter decomposition in my country, and the majority of these studies concentrate on the quality of the litter matrix and the release dynamics of physicochemical elements (Jia et al., 2016). Given the strict stoichiometric requirements for microbial decomposer growth, it is possible that the scarcity of a particular nutrient will slow down the decomposition of litter (Ni et al., 2021). Numerous studies have discovered a strong correlation between the rate of litter decomposition and the initial N content of the litter as well as N-related substrate quality indicators. Indicators of C/N and C/P are frequently used to forecast the rate at which litter will decompose. The decomposition rate decreases as C/N and C/P values rise (Aber and Melillo, 1982; Magill et al., 2000; Sariyildiz and Anderson, 2003). It is consistent with the findings of this research (Table 1). The same pattern was shown in Figure 5. The decomposition rate of mixed litter was found in this study to be significantly higher than that of coniferous forest, but lower than that of pure broad–leaved forest. This is consistent with the findings of Pastor et al. (1984) who conducted experiments on nitrogen mineralization and litter decomposition in pine forests on Blackhawk Island in the United States. Sariyildiz also came to the same conclusion in his simulated experiments on beech and oak trees (Sariyildiz, 2015).

The primary factor influencing nutrient release is plant litter quality, and its C/N value is frequently regarded as an important attribute for measuring litter quality (Silver and Miya, 2001; Hu et al., 2006). For instance, Brady and Weil (1996) discovered that nitrogen release occurs when C/N values in the remaining litter are lower than 25 and nitrogen fixation occurs when C/N values are >25 (Killham, 1994). The rate of decomposition can be regulated by early litter decomposition C/N (Cotrufo and Ineson, 1995; Berg, 2000). Litter C/P has a threshold between 200 and 480, and this threshold affects how much litter P content is released. The litter P content is a net release when the litter C/P is <480 (Gosz et al., 1973; Dziadowiec, 1987; Manzoni et al., 2010). Furthermore, Liu’s research made the case that litter N/P can be used as a gauge for determining nutrient limitation. P limit how much litter can decompose if N/P is >25 (Liu et al., 2016). The results of this experiment demonstrated that the phenomenon that P limited the decomposition of coniferous species could very well be improved by the addition of broad–leaved species. In conclusion, adding broad–leaved litter can increase the rate at which coniferous litter decomposes, this is because mixed litter decomposes more quickly than litter from a single tree species.

Previous research has shown that single-species litter and mixed litter have significantly different community compositions and structures (Aneja et al., 2006; Chapman and Newman, 2010; Prescott and Grayston, 2013; Santonja et al., 2017). Microbial biomass and community composition can vary depending on the microenvironment or chemical makeup of the litter (Malosso et al., 2004; Das et al., 2007; Xu et al., 2013). The current study discovered differences between the alpha diversity of single-species litter and mixed litter microbial communities. Mixed litter made up of *Populus × canadensis* and *Pinus sylvestris* var. *mongolica* differed significantly from *Pinus sylvestris* var. *mongolica* litter in terms of bacterial richness, diversity, uniformity and coverage, but not from *Populus × canadensis* litter. However, the coverage and richness of mixed litter and *Populus × canadensis* litter were not significantly different, and the fungal community diversity and homogeneity of the *Populus × canadensis* – *Pinus sylvestris* var. *mongolica* litter were only significantly higher than those of *Populus × canadensis*. This has also been supported by earlier researches (Aneja et al., 2006; Chapman and Newman, 2010; Prescott and Grayston, 2013; Santonja et al., 2017). Proteobacteria and Ascomycetes are the most abundant taxa in the early stage of litter decomposition and are the main decomposers (Voříšková and Baldrian, 2012; Zhang et al., 2017). This study also found that Proteobacteria dominated the bacterial community and Ascomycota dominated the fungal community. According to research, fungi and bacteria work together to speed up the decomposition of litter (Wright and Covich, 2005). Fungi are primarily responsible for the decomposition of carbonaceous organic matter (Frey et al., 1999; Guggenberger et al., 1999; Pascoa and Cássio, 2004), while bacteria primarily consume nitrogenous organic matter (Six et al., 2002). Ascomycetes, the predominant decomposing bacteria in the three types of litter, are primarily in charge of breaking down cellulose and hemicellulose (Pointing and Hyde, 2000; Sánchez, 2009; Zhang et al., 2016), and Proteobacteria are in charge of breaking down proteins (Schweitzer et al., 2001; Kazakov et al., 2009).

In the breakdown of apoplast, various genera also play various roles. For instance, the top 10 species in terms of relative abundance in this study, *Massilia*, *Allorhizobium-Neorhizobium-Parararhizobium-Rhizobium*, *Actinoplanes*, *Devosia*, *Flavobacterium*, and *Microbacterium*, all have the job of breaking down organic acids, polyols, aromatic compounds, and other substances in the environment (Du et al., 2011; Feng et al., 2019; Menon et al., 2019). Both simple carbohydrates and refractory materials are thought to be capable of being broken down by *Alternaria* (Jatav et al., 2020). An endophytic fungus called *Paraconiothyrium* degrades lignin (Gao et al., 2011). They all play a role in the breakdown of *apoplastic* matter, which is consistent with the study’s findings.

The structure and operation of the litter microbial community are also impacted by the differences between coniferous and broad-leaved litter’s physical and chemical characteristics. Previous researches have also demonstrated a connection between the chemical properties of decomposed substrates and the diversity of the microbial community (Hawke and Vallance, 2015; Zeng et al., 2017). The two main microbial taxa involved in decomposition litter are fungi and bacteria, and depending on the ecosystem and type of litter, their functional characteristics and carbon requirements may vary (Lin et al., 2018). Although fungi are thought to decompose more quickly than bacteria, bacteria are more effective at using labile carbon compounds (Hunt et al., 1987). This may account for the stronger relationship between apomictic Dw and bacterial community alpha diversity as well as the lack of a relationship with fungal communities. When compared to coniferous pure forest litter, mixed forest litter has different carbon and mineral nutrient contents, offering a wider substrate for microbes that break down organic matter (Hooper and Vitousek, 1998; Hättenschwiler et al., 2011).

## Conclusion

In this study, it was discovered that the decomposition effect of mixed litter was different from that of a species of tree by simulating the mixed decomposition of *Populus × canadensis* Moench and *Pinus sylvestris* var. *mongolica* litter. (1) Compared to leaf litter from a single tree species, mixed leaf litter had different nutrients. However, the contents of total P, C/N, N/P and C/P were as YZ was between YS and ZS. The total C and total N of YZ were higher than those of single tree species. (2) There were variations in the organization and composition of the microbial communities in mixed leaf litter compared to of single tree species, but the difference was not very significant. (3) The investigation revealed that leaf nutritional variables had an impact on the variety of the microbial community composition and organization in leaf litter. (4) The decomposition of *Populus × canadensis* Moench litter was significantly aided by the addition of *Pinus sylvestris* var. *mongolica* litter. N/P, C/P, total N, and total P had a substantial impact on the bacterial community, while C/N, total C, and total N had a large impact on the fungal community. Consequently, this research provided a theoretical framework for the investigation of the decomposition mechanism of mixed litter from the nutritional level and microbial community.

## Data availability statement

The datasets presented in this study can be found in online repositories. The names of the repository/repositories and accession number(s) can be found at: https://www.ncbi.nlm.nih.gov/, PRJNA832892.

## Author contributions

WZu designed the research. JL, CD, and WZa performed the research. JL, CD, YW, and YZ analyzed the data. WZu, CD, and JL wrote the manuscript. All authors contributed to the article and approved the submitted version.

## Funding

This work was supported by the National Key Research and Development Program of China (grant number 2021YFD2201205), the Basic Research Fund of RIF (grant no. CAFYBB2020SZ002), the National Natural Science Foundation of China (grant nos. 31870662 and 32271843), and Liaoning Province Scientific Research Funding Project (LSNQN202012).

## Conflict of interest

The authors declare that the research was conducted in the absence of any commercial or financial relationships that could be construed as a potential conflict of interest.

## Publisher’s note

All claims expressed in this article are solely those of the authors and do not necessarily represent those of their affiliated organizations, or those of the publisher, the editors and the reviewers. Any product that may be evaluated in this article, or claim that may be made by its manufacturer, is not guaranteed or endorsed by the publisher.

## Supplementary material

The Supplementary material for this article can be found online at: https://www.frontiersin.org/articles/10.3389/fmicb.2022.1009091/full#supplementary-material

## Footnotes

^1^ http://www.arb-silva.de

^2^ https://www.genescloud.cn
