# Supplementary figures and images for: Litter mixing promoted decomposition rate through increasing diversities of phyllosphere microbial communities
Source: Front Microbiol. 2022 Nov 8;13:1009091. doi: 10.3389/fmicb.2022.1009091 (PMC9678933; doi:10.3389/fmicb.2022.1009091)

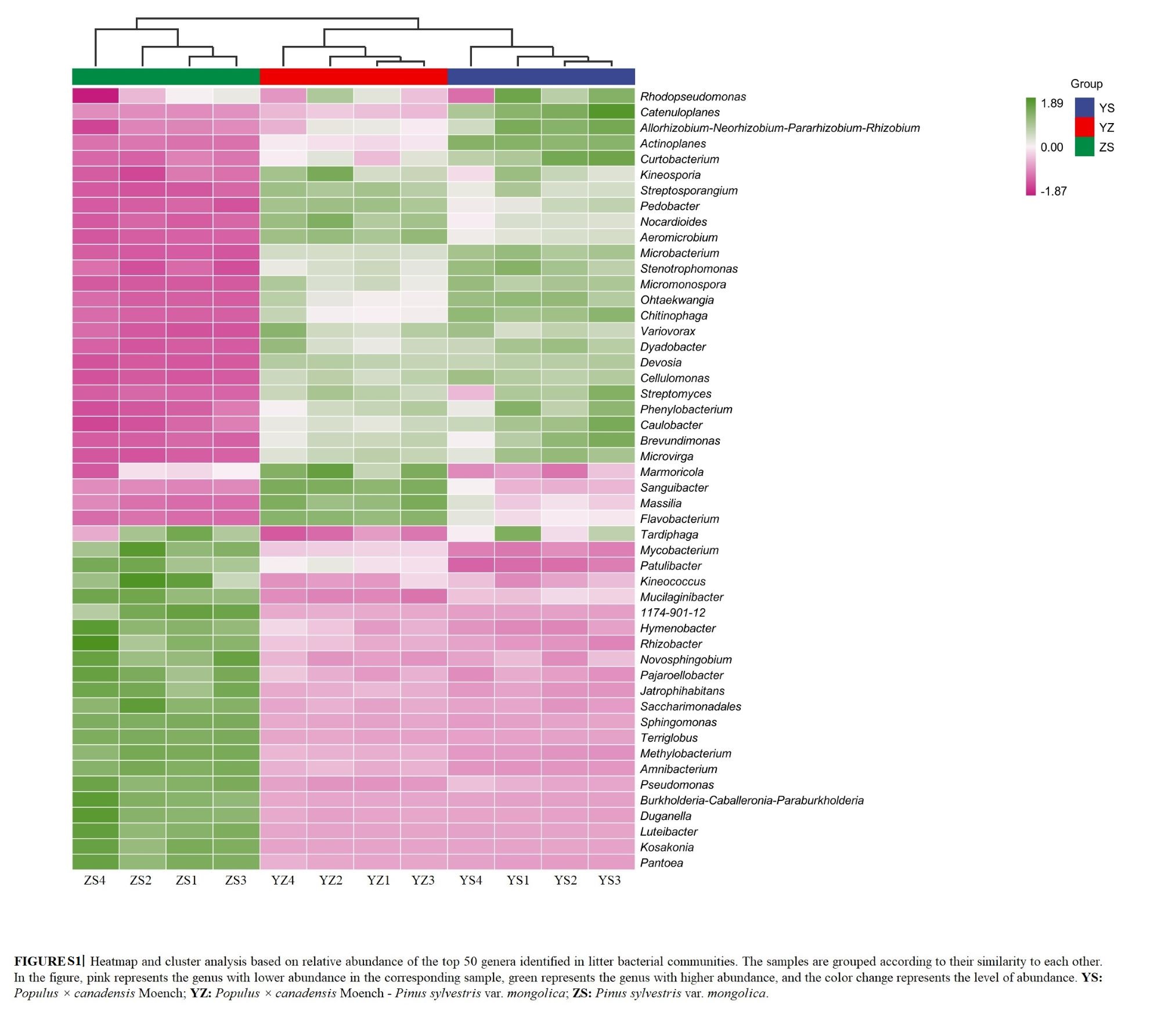

Supplement: Supplementary file 1 [file Image_1.JPEG]

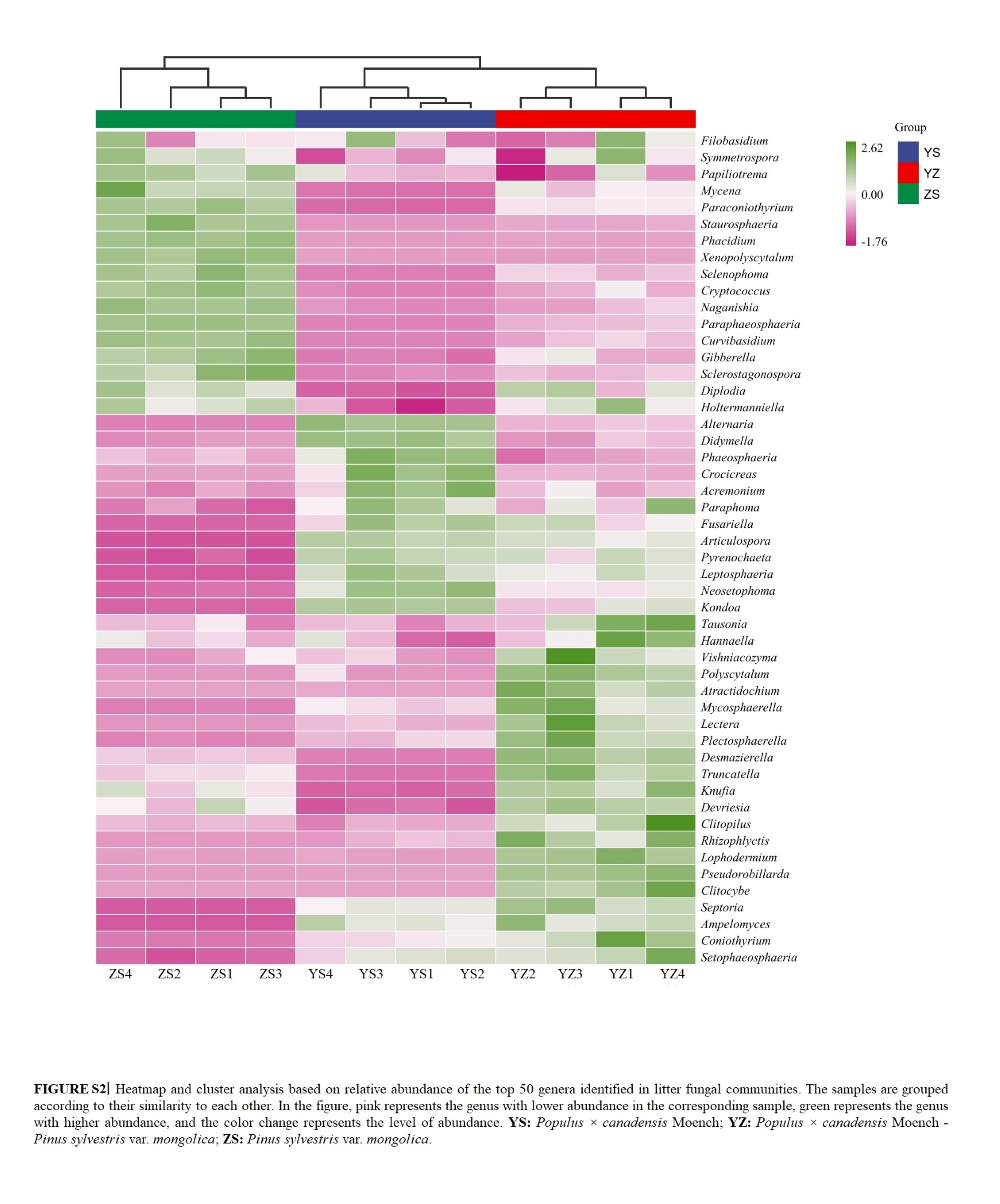

Supplement: Supplementary file 2 [file Image_2.JPEG]
